# Proceedings Veterinary Medical Societies

**Published:** 1886-10

**Authors:** 


### PROCEEDINGS VETERINARY MEDICAL SOCIETIES.

The annual meeting of the United States Veterinary Medical Association, held at the Rossmore Hotel, New York, on Tuesday, September 21st., furnished material for a great deal of reflection to all who have a sincere interest in the welfare of the Association and of the Veterinary profession at large. With the ample facilities for society meetings furnished by the Medical Societies and Veterinary Schools in New York, it seems unfortunate that a hotel should have been chosen for the assembly, and one too, where the clerk's conception of the meeting was, "that crowd who are dining."

The morning was occupied by the meeting of the Comitia Minora and the regular session was not called until an hour after the time announced. The meeting was opened by the President, Prof. L. McLean, with a short, concise address of welcome and review of his year's presidency. He called attention to Article 2, of the constitution, which defines the object of the meetings, and added a truthful criticism of the want of interest, and indifference displayed by many members of the profession. He sarcastically derided the cry that the veterinary profession needs elevation, while he regretted the fact that many members of it do. Calling attention to the numerous subjects, particularly some of the contagious diseases, which might be brought forward and discussed with advantage both to the members and to the public at large, he hoped that the future of the association would retrieve some of the neglect of the past. The reading of the minutes included those of the previous annual meeting and those of the semi-annual meeting held in Boston last March, the legality of the latter was questioned owing to the neglect of the Secretary in not giving the proper notice. The effects of this error were plainly seen in the absence of the dozen active members who usually represent Massachusetts.

The "Special Committee to secure a uniform standard of examinations by the different Veterinary Colleges of North America," furnished a report through the chairman announcing but mediocre progress, and suggesting that means be afforded the committee to visit the various schools in order to confer with the authorities personally.

While recognizing the importance of the subject, we do not believe it to be feasible. The schools are independent of government direction; some are private institutions; while others are controlled by University charters whose constitution would forbid any compact with other schools.

What is practicable is for the United States Veterinary Medical Association, the representative body of veterinarians in America, to define the *minimum* curriculum which any veterinary school must enforce in order to be recognized by the Association, and in order to render its graduates eligible to membership. This would not in any way conflict with the eligibility of the many honorable non-graduate practitioners, whose entry into the profession antedates the present opportunity for obtaining a regular education, and the admission of non-graduates should be limited to these.

The report of the committee on Diseases showed a melancholy lack of interest, which was only compensated for by an able review from the chairman, Prof. Liautard, defining the contagious diseases which need special attention, and the methods to be used in reporting them. The Prize Committee failed to make a report. The two papers which had been presented for competition (both on Parturient Apoplexy, and already published in the *American Veterinary Review*), were read by the Secretary, and by a large majority the prize was awarded to paper No. 1, which proved to be the production of Dr. T. S. Butler, of Chillicothe, Ohio.

Some twelve new members were added to the Association, and the election of officers for the ensuing year, resulted in the choice of President, Prof. A. Liautard, New York; Vice-President, Prof. William A. Zuill, Philadelphia; Secretary, Prof. C. B. Michener, New York; Treasurer, Prof. J. L. Robertson, New York. H.

